# Descriptive Analysis of COVID-19 Patients Who Required Endoscopic Evaluation for Gastrointestinal Bleeding

**DOI:** 10.7759/cureus.81241

**Published:** 2025-03-26

**Authors:** Gianmarino Gianfrate, Breanna Gillie, Charles Renner, Brian Gruber

**Affiliations:** 1 General Surgery, St Elizabeth Youngstown Hospital, Youngstown, USA; 2 Vascular Surgery, University of Kentucky College of Medicine, Lexington, USA; 3 Trauma Surgery, St Elizabeth Youngstown Hospital, Youngstown, USA

**Keywords:** acute gastrointestinal bleed, colonoscopy, covid-19, egd, endoscopy, esophagogastroduodenoscopy (egd), morbidity and mortality, sars-cov-2 infection, colonoscopy

## Abstract

Introduction: Gastrointestinal (GI) hemorrhage has been reported in patients with SARS-CoV-2. Although there is consensus that the infection is associated with GI sequelae, controversy remains regarding its clinical significance. Endoscopic intervention was limited during the pandemic due to safety concerns and resource constraints, which may have hindered a full assessment of the impact of GI hemorrhage on patient outcomes. This paper aims to evaluate the outcomes of patients diagnosed with SARS-CoV-2 and concurrent clinically significant GI hemorrhage.

Materials and methods: A total of 125 patients (69 male, 56 female) over the age of 18, with signed procedural consent, were included. All met the criteria for a SARS-CoV-2 diagnosis and underwent diagnostic endoscopic intervention. Data were analyzed using the Mann-Whitney U test with Excel (Microsoft Corporation, Redmond, WA, USA) and SPSS Statistics version 25 (IBM Corp., Released 2017. IBM SPSS Statistics for Windows, Version 25.0. Armonk, NY: IBM Corp.).

Results: The overall hospital length of stay was 8 ± 6 days. A subset analysis compared patients requiring ICU admission with those who did not. The average ICU length of stay was 13 ± 6 days, compared to 5 ± 3 days for non-ICU patients. Among patients who underwent esophagogastroduodenoscopy, 65% (70/108) required intervention, while 16% (3/19) of colonoscopies required intervention. There was no significant difference in underlying comorbidities or rates of non-invasive mechanical ventilation between groups. Overall mortality was 50% (62/125), with no significant difference between ICU (26/50) and non-ICU (36/75) patients (52% vs. 48%).

Conclusions: While studies have indicated an increased risk of GI complications in SARS-CoV-2 patients, many have not differentiated between hemorrhagic and non-hemorrhagic sequelae or accounted for the level of care. We conclude that there was higher mortality among patients requiring endoscopic intervention, regardless of their level of care or patient-specific factors.

## Introduction

Research into SARS-CoV-2, the virus responsible for the COVID-19 pandemic, is ongoing as investigations into its short- and long-term sequelae evolve. According to the World Health Organization, there have been 754,018,841 cumulative confirmed cases globally, resulting in 6,817,478 deaths, with the United States contributing 100,941,827 cumulative cases and 1,097,246 deaths [1]. According to the literature, the COVID-19 pandemic caused a significant reduction in emergency surgical operations and overall admissions to emergency departments due to widespread hospital-related fear and anxiety experienced by most patients during the peak of the outbreak [1,2].

While SARS-CoV-2 primarily causes pulmonary disease, numerous extrapulmonary manifestations have also been described, affecting the cardiac, gastrointestinal (GI), dermatologic, neurologic, hepatobiliary, and hematologic systems [2-4]. These extrapulmonary sequelae increase disease complexity and impact patient morbidity and mortality [2,3]. Gaining a deeper understanding of these extrapulmonary manifestations is essential for determining appropriate levels of care, understanding the disease course, and minimizing negative patient outcomes.

This paper aims to further investigate the relationship between SARS-CoV-2 and specific GI and hematologic sequelae, particularly their role in increasing morbidity and mortality due to GI hemorrhage.

The GI manifestations of SARS-CoV-2 that have been reported include nausea, vomiting, diarrhea, abdominal pain, anorexia, elevated hepatic markers, elevated bilirubin, diminished albumin, and GI hemorrhage [2-5]. These sequelae have been observed in 12-61% of patients who tested positive for SARS-CoV-2, although their association with increased mortality remains controversial in the literature [2,3,6,7]. The prevalence of GI manifestations appears to be higher in the United States than in the global population [8-10].

The pathophysiology underlying these GI manifestations appears to be multifactorial. Early evidence suggested that the expression of angiotensin-converting enzyme 2 (ACE-2) on intestinal mucosal cells supported the possibility of fecal-oral transmission of SARS-CoV-2 [2,8,11]. This expression has also been proposed as a potential mechanism of virus-mediated direct tissue injury to the intestinal epithelium [11,12]. This is further supported by evidence demonstrating live viral shedding in fecal studies and histopathologic findings of endothelial inflammation and lymphocytic infiltration in the submucosa of the GI tract in infected individuals [8,11,13].

Given this propensity for direct GI epithelial damage, consideration of the hematologic impacts of SARS-CoV-2 becomes important when investigating GI sequelae. Although a hypercoagulable state is common in critical care patients, COVID-associated coagulopathy was highlighted in early research as a possible cause of increased mortality and morbidity in infected patients [5,14-16]. Early data supported hypercoagulability due to increased ACE-2 expression on endothelial cells in infected individuals, resulting in endothelitis and an increased thrombotic risk [17-19].

Thrombin generation assays and rotational thromboelastometry analyses conducted on SARS-CoV-2-infected patients provided evidence supporting a hypercoagulable state. However, consumptive coagulopathy was observed when comparing D-dimer, prothrombin time, antithrombin III, and platelet concentrations in the same group [14,19]. As the pandemic progressed, the coagulopathy associated with SARS-CoV-2 was shown to be as prevalent as thrombosis [5,20]. Many patients exhibited a significantly increased bleeding risk, with pathophysiology closely resembling disseminated intravascular coagulation and other infectious hemorrhagic diseases such as Lassa virus, Ebola, and hemorrhagic dengue [5,14,15,20].

As a result of the evolving understanding of COVID-19 pathophysiology, early studies reported increased survival in patients receiving therapeutic anticoagulation compared to those who did not, leading to recommendations for early anticoagulation in critically ill patients [5,14,19]. As research progressed, recommendations evolved as well. The current recommendation for thrombotic prophylaxis remains routine risk assessment for hospitalized SARS-CoV-2 patients, with prophylaxis indicated for those without absolute contraindications. However, prophylaxis continues to be routinely used in critical care settings even without confirmed thrombosis [8,16,18]. The significance of this lies in the fact that SARS-CoV-2 infection may more frequently result in consumptive coagulopathy and an increased risk of bleeding due to virus-induced coagulopathy, direct viral damage to cells, therapeutic anticoagulation administration, or a combination of these factors.

The annual health expenditure for managing GI disease is $135.9 billion [21]. Given the economic strain and resource limitations placed on the healthcare industry throughout the pandemic, an increase in GI bleeding can be considered a significant burden on the population. GI hemorrhage has been reported in 0.9-13% of patients hospitalized with COVID-19, the majority of whom were found to have duodenal and gastric ulcerations [22-26]. Although the incidence of GI hemorrhage in patients with SARS-CoV-2 is well documented in the literature, controversy remains as to whether this represents an increase from previous years [2,3,13].

While the recommendation for significant GI hemorrhage remains endoscopic evaluation within 24 hours of presentation in those with a Glasgow-Blatchford score >6, which corresponds to >50% mortality, it appears that in the initial stages of COVID-19, endoscopic intervention was not routinely undertaken [2,3,8,27]. Previously proposed explanations for this include resource limitations and the initial fear of an increased risk of bidirectional transmission in endoscopy suites [23,28,29]. Other potential reasons include the respiratory stability of these patients and the availability of prophylactic protective equipment for endoscopy staff [23,29].

Studies have since shown that endoscopic procedures pose minimal risk for viral transmission, and resource availability has improved [30]. Given the alleviation of these factors and the ongoing debate within the literature regarding the clinical significance of SARS-CoV-2-related GI hemorrhage, this paper aims to explore the characteristics of COVID-19 patients who qualified for endoscopic intervention for GI hemorrhage and to assess the impact of this sequela on outcomes, including length of hospital stay, ICU length of stay, morbidity, and mortality in our patient population.

## Materials and methods

We performed a retrospective, single-center cohort study in which we reviewed all inpatient endoscopic procedures performed on SARS-CoV-2-infected patients with clinical or laboratory concerns for GI hemorrhage. The Institutional Review Board of Mercy Health Youngstown, LLC, issued approval 21-018, and a Health Insurance Portability and Accountability Act waiver was obtained prior to the start of the study. All data, including endoscopy results, length of hospital stay, length of ICU stay, requirement for blood products, and time to hospital discharge, were reviewed from January 2020 through February 2021 at St. Elizabeth Hospital in Youngstown, Ohio, USA. The data cutoff was February 1, 2021.

The confirmation of SARS-CoV-2 infection included laboratory confirmatory testing and clinical diagnosis by board-certified infectious disease specialists employed at our facility within five weeks of endoscopic intervention. Laboratory-confirmed cases were initially diagnosed in 2020 by pharyngeal swab and later by viral polymerase chain reaction (PCR) testing as diagnostic tools evolved throughout the pandemic.

Patient selection criteria

Diagnostic Measures

Patients included in the study were 18 years of age or older and had signed consent either themselves or through a legal guardian for endoscopic procedures. Diagnosing SARS-CoV-2 infection first required high clinical suspicion, as guided by the World Health Organization interim recommendations [1]. Once a case was suspected, a pharyngeal swab for SARS-CoV-2 was performed. Those who received a positive result were then deemed SARS-CoV-2 positive.

In early 2020, given the uncertain sensitivity of pharyngeal swabs and the potential for inter-provider sampling error, patients with high clinical suspicion for SARS-CoV-2 who tested negative via pharyngeal swab received early consultation with infectious disease specialists. According to their expert opinion, these specialists could make a clinical diagnosis despite negative testing based on chest CT, chest X-ray, or clinical manifestations of infection (fever, respiratory symptoms, leukocytosis with lymphocyte predominance).

With the advent and evolution of reverse transcriptase PCR (RT-PCR) testing for SARS-CoV-2, patients evaluated later in this period who had a negative pharyngeal swab underwent confirmatory RT-PCR testing. Patients included in our study’s data collection were those with a positive pharyngeal swab, a positive RT-PCR test, or a clinical diagnosis made by infectious disease specialists within our institution.

All intubated patients included in the study were on H2 blockers or proton pump inhibitors for GI prophylaxis on the day of intubation. There were no relevant exclusion criteria in this study; however, none of the patients had a history of prior foregut surgery.

Comorbidities and Coexisting Illness Measures

Patients in this study were evaluated for coexisting illnesses and prior SARS-CoV-2 infection before their hospital admission. Comorbidities were subdivided by organ system, and data were categorized into the following groups: previous cardiac disease (atrial fibrillation, congestive heart failure, or prior myocardial infarction); chronic kidney disease (diagnosed by a nephrologist at least six months before diagnosis); chronic lung disease (diagnosed by a pulmonologist or confirmed by a positive pulmonary function test); peripheral or cerebrovascular disease (including transient ischemic attack, stroke, deep vein thrombosis, pulmonary embolism, vasculitis, or venous insufficiency); diabetes mellitus (HbA1c of 6.4% or greater); liver disease (biopsy-proven cirrhosis); and hypertension (systolic pressure over 140 mmHg requiring at least one blood pressure medication).

Endoscopic Intervention

All endoscopy staff were equipped with and required to wear appropriate personal protective equipment, as outlined by the Centers for Disease Control guidelines, including protective suits, face shields, N95 masks, and surgical gloves to limit the risk of bidirectional exposure. All inpatient esophagogastroduodenoscopies and colonoscopies performed on diagnosed patients with suspected GI bleeding were included in the data collection. All procedures were conducted for the localization, control, and evaluation of suspected GI bleeding and not for viral RNA detection via biopsy, as the necessary resources for biopsied viral RNA detection were never readily available at our facility.

Statistical Analysis

All statistical analyses were conducted using Excel (Microsoft Corporation, Redmond, WA, USA) and the Mann-Whitney U test in SPSS Statistics version 25 (BM Corp., Released 2017. IBM SPSS Statistics for Windows, Version 25.0. Armonk, NY: IBM Corp.). Variables are expressed as mean ± standard deviation (SD) and were analyzed accordingly. Only data from SARS-CoV-2 positive patients who underwent inpatient endoscopy were analyzed.

## Results

A total of 125 patients (69 men, 56 women) were included, with an average age of 70 ± 14 years (Table 1).

**Table 1:** Demographics and baseline features of 125 patients infected with SARS-CoV-2 SARS-CoV-2: severe acute respiratory syndrome coronavirus 2, ICU: intensive care unit, BiPAP: bilevel positive airway pressure, CPAP: continuous positive airway pressure, EGD: esophagogastroduodenoscopy

Variable	All patients (n=125)
Age (year)	70 + 14
Men	69 (55%)
Women	56 (45%)
Caucasian	87 (70%)
African American	28 (22%)
Other race	10 (8%)
Length of stay (days)	8 + 6
ICU admission	50 (40%)
Tobacco use (current or within 15 years, minimum of ¼ pack per day)	78 (62%)
Alcohol use (minimum of three beers or equivalent)	106 (85%)
Patients on blood thinner/anti-platelet medication prior to arrival	59 (47%)
Blood thinner/anti-platelet medication reversed within 24 hours of admission (n=59)	26 (44%)
Positive pressure ventilation (BiPAP, CPAP, mechanical ventilation)	107 (86%)
EGD	106 (84.8%)
Colonoscopy	17 (14%)
EGD and colonoscopy	2 (2%)
EGD intervention (n=108)	70 (65%)
Colonoscopy intervention (n=19)	3 (16%)
Requirement of blood products (n=57)	57 (46%)
Packed red blood cells (n=57)	57 (100%)
Fresh frozen plasma (n=57)	17 (30%)
Platelets (n=57)	6 (10%)
Modified massive transfusion protocol (n=57)	5 (9%)
ICU admission	50 (88%)
Coexisting illness	100 (80%)
More than one coexisting illness	62 (62%)
Cardiac disease	32 (32%)
Peripheral or cerebrovascular disease	17 (17%)
Chronic lung disease	22 (22%)
Chronic kidney disease, diabetes mellitus	4 (4%), 34 (34%)
Hypertension	51 (51%)
Biopsy-proven cirrhosis	2 (2%)
All-cause mortality	62 (50%)

Among these patients, the majority were Caucasian individuals (87/125, 70%) and African American individuals (28/125, 22%), with a small population consisting of Native American, Asian, and Pacific Islander individuals (10/125, 8%) (Table 1). Within our sample, the mean length of hospital stay was 8 ± 6 days (Table 1). Most patients had pre-existing comorbidities requiring medication, as well as socioeconomic factors that may have contributed to symptom severity, length of hospital stay, and disease outcomes. Pre-existing comorbidities were found in 100/125 (80%) of patients, with the most prevalent being hypertension (51/100, 51%), diabetes mellitus (34/100, 34%), and cardiac pathologies (32/100, 32%). Additionally, among those with pre-existing comorbidities, 62/100 (62%) had more than one (Table 1).

A total of 78/125 (62%) patients were either current smokers or had smoked within the past 15 years, while 108/125 (86%) reported current alcohol use. Although specific consumption rates were not quantified, only 2/125 (2%) had a history of biopsy-proven liver cirrhosis (Table 1). Anticoagulation use prior to admission was noted in 59/125 (47%) of patients, including those taking clopidogrel, apixaban, warfarin, aspirin 325 mg, ticagrelor, and rivaroxaban. Of these, 26/59 (44%) required anticoagulant reversal within the first 24 hours of admission (Table 1).

To determine the effect of the level of care on patient outcomes, a subset analysis was performed, separating those requiring ICU admission from those not requiring ICU admission (Table 2). The average hospital length of stay among ICU patients was 13 ± 6 days, compared to 5 ± 3 days for non-ICU patients. Both populations had more men than women, with the predominant race being Caucasian individuals (Table 2). ICU patients had a higher percentage of pre-admission anticoagulant and anti-platelet use (40% vs. 25%), as well as pre-existing comorbidities (96% vs. 69%) (Table 2).

**Table 2:** Demographics and baseline features of ICU and non-ICU patients infected with SARS-CoV-2 ICU: intensive care unit, SARS-CoV-2: severe acute respiratory syndrome coronavirus 2

Variable	Non-ICU patients (n=75)	ICU patients (n=50)	Mann-Whitney U test (p<0.05)
Age (year)	68 + 14	72 + 16	-
Male	41 (55%)	28 (56%)	0.90448
Female	34 (45%)	22 (44%)	0.90448
Caucasian	42 (56%)	45 (90%)	0.00132
African American	23 (31%)	5 (10%)	0.05118
Other race	10 (13%)	0	0.20766
Length of stay (days)	5 + 3	13 + 6	-
ICU length of stay	-	8 + 6	-
Tobacco use (current or within 15 years, minimum of ¼ pack per day)	58 (77%)	20 (40%)	0.00042
Alcohol use (minimum of three beers or equivalent)	61 (81%)	45 (90%)	0.4122
Patients on blood thinner/anti-platelet medication prior to arrival	19 (25%)	40 (80%)	<0.00001>
Blood thinner/anti-platelet reversed within 24 hours of admission	0	26 (52%)	<0.000001>
Coexisting illness	52 (69%)	48 (96%)	0.01174
More than 1 coexisting illness	38 (38%)	24 (48%)	0.90448
Cardiac disease	19 (25%)	13 (26%)	0.8493
Peripheral or cerebrovascular disease	10 (13%)	7 (14%)	0.95216
Chronic lung disease	10 (13%)	12 (24%)	0.37886
Chronic kidney disease	4 (5%)	0	-
Diabetes mellitus	20 (27%)	14 (28%)	0.90448
Hypertension	33 (44%)	18 (36%)	0.45326
Liver disease	2 (3%)	0	-
Requirement of blood products for control of hemorrhage (n=57)	2 (4%)	50 (88%)	<0.00001>

A total of 107/125 patients (86%) required some form of positive pressure respiratory support during their admission, including 57/75 (76%) non-ICU patients and 48/50 (96%) ICU patients (Tables 1-2). A substantial portion of ICU patients, 28/50 (56%), progressed to invasive mechanical ventilation (Table 2). The mean ICU length of stay was 7.7 ± 6 days, with the longest ICU stay being 31 days (Table 2). Mortality within the entire sample was 62/125 (50%), with a greater proportion among those requiring ICU admission (26/50, 52%) compared to non-ICU admissions (36/75, 48%) (Tables 1-2).

All patients reviewed met the criteria for diagnostic endoscopy during their admission. Of these, 106/125 (85%) underwent esophagogastroduodenoscopy (EGD), 17/125 (14%) underwent colonoscopy, and 2/125 (2%) underwent both EGD and colonoscopy (Table 1). Among those who underwent EGD, 75/108 (69%) required intervention to control GI hemorrhage, including epinephrine injection, clip placement, or both (Table 3). Most patients with hemorrhage amenable to intervention had bleeding gastric or duodenal ulcerations. Intervention with injection or clipping to control hemorrhage was required in 3/19 (16%) colonoscopies performed (Table 3). GI hemorrhage significant enough to require blood product transfusion was observed in 57/125 (46%) patients, of whom 50/57 (88%) were admitted to an ICU (Table 3). This subset's 5/57 (9%) required a massive transfusion protocol, including at least 3 units of RBCs, 3 units of FFP, and 1 unit of platelets (Table 1).

**Table 3:** Interventions and clinical outcomes for ICU and non-ICU patients ICU: intensive care unit, EGD: esophagogastroduodenoscopy

Variable	Non-ICU (n=75)	ICU (n=50)	Mann-Whitney U test (p<0.05)
Intubated	0	28 (56%)	<0.00001>
Bilevel positive airway pressure/continuous positive airway pressure	57 (76%)	48 (96%)	0.05876
Colonoscopy	17 (23%)	2 (4%)	0.0784
Percent of total colonoscopy (N=19)	17/19 (89.5%)	2/19 (10.5%)	-
Colonoscopy intervention to control hemorrhage	3/17 (17.6%)	0/2 (0%)	-
EGD	60 (80%)	48 (96%)	0.13104
Biopsy	58 (77%)	45 (90%)	0.23404
Endoscopic Intervention (epinephrine or clip placement)	45 (60%)	25 (50%)	0.34722
Mild gastritis or duodenitis	55 (73%)	45 (90%)	0.10100
Severe gastritis or duodenitis	5 (7%)	3 (6%)	0.95216
1 ulcer	25 (33.3%)	15 (30%)	0.75656
2+ ulcer	10 (13.3%)	5 (10%)	0.75656
Bleeding vessel	10 (13.3%)	7 (14%)	0.95216
Discharge to home or skilled nursing facility	39 (52%)	24 (48%)	0.77
All-cause mortality	36 (48%)	26 (52%)	0.75656

Within our sample, a greater proportion of patients requiring ICU admission reported a history of alcohol use compared to non-ICU patients (45/50 or 90% vs. 61/75 or 81%). In contrast, a lower proportion of ICU patients reported a history of tobacco use (20/50 or 40% vs. 58/75 or 77%) (Table 2). The prevalence of comorbidities was higher in the ICU cohort than in the non-ICU cohort (48/50 or 96% vs. 52/75 or 69%), as was the presence of multiple comorbidities (24/50 or 48% vs. 38/75 or 38%) (Table 2).

Liver disease, hypertension, and chronic kidney disease were reported more frequently among non-ICU patients (2/75 or 3%, 33/75 or 44%, and 4/75 or 5%, respectively) compared to ICU patients (0%, 18/50 or 36%, and 0%, respectively) (Table 2). However, chronic lung disease was more prevalent in the ICU group than in the non-ICU group (12/50 or 24% vs. 10/75 or 13%) (Table 2). The prevalence of pre-existing heart disease was similar between ICU and non-ICU patients (13/50 or 26% vs. 19/75 or 25%), as was the prevalence of pre-existing peripheral vascular disease and cardiovascular disease (7/50 or 14% vs. 10/75 or 13%) and pre-existing diabetes (14/50 or 28% vs. 20/75 or 27%).

There were no significant differences in the rates of non-invasive mechanical ventilation between those requiring ICU-level care and those who did not (U=0.05876). Additionally, there was no significant difference in the presence of underlying comorbidities between ICU and non-ICU patients (U=0.1174). While no statistically significant differences were found between the cohorts, trends in the data indicated that ICU patients tended to have a higher prevalence of mild gastritis or duodenitis compared to non-ICU patients (45/50 or 90% vs. 55/75 or 73%), with a relatively equal number of severe cases between the groups (Table 3).

ICU patients had a similar number of identifiable ulcerations and bleeding vessels on diagnostic endoscopy compared to non-ICU patients. A single ulcer was reported in 25/75 (33%) of non-ICU patients compared to 15/50 (30%) of ICU patients. Multiple ulcerations were observed in 10/75 (13%) of non-ICU patients compared to 5/50 (10%) of ICU patients. The presence of a bleeding vessel was documented in 10/75 (13%) of non-ICU patients and 7/50 (14%) of ICU patients (Table 3). Of these, 45/75 (60%) of non-ICU patients required endoscopic intervention to control hemorrhage, compared to 25/50 (50%) of ICU patients (Table 3).

There were no statistically significant differences between the groups, as outlined in Table 3. When comparing overall mortality between groups, no significant differences were observed between ICU and non-ICU patients (U=0.75656).

## Discussion

The GI manifestations of SARS-CoV-2 have been well documented in the literature throughout the pandemic. Specifically, the rate of GI hemorrhage has increased from 1.1% to 13% since the onset of COVID-19 [25]. However, there is ongoing debate regarding the clinical significance of this increase and the appropriate management strategies. Early studies comparing conservative management of GI hemorrhage to the current standard of early endoscopic intervention found that conservative measures were equally effective in controlling hemorrhage [7]. Martin et al. reported no significant difference in outcomes between patients with GI hemorrhage treated with endoscopy and those managed conservatively with medical therapy [24,31]. Similarly, a study conducted in China early in the pandemic found no significant 30-day morbidity or mortality increase with conservative management [23]. Another study demonstrated no significant differences in outcomes for patients followed for six months after discharge [28]. These studies primarily focused on patients outside the critical care setting who presented with mild GI manifestations of SARS-CoV-2 and subsequently tested positive.

In our study, we aimed to further stratify our sample into patients requiring ICU-level care and those who did not, to assess whether the level of care impacted patient outcomes. Specifically, we examined only those infected with SARS-CoV-2 who experienced clinically significant GI hemorrhage and qualified for endoscopic intervention, both in critically ill and non-critically ill patients.

A significant decrease in global endoscopic evaluation of GI hemorrhage was reported throughout the pandemic, despite current recommendations that all new GI bleeding undergo EGD within 24 hours of presentation [24,30]. A decline of more than 50% in endoscopic interventions has been documented during the pandemic when compared to similar periods in prior years [7,17,31,32]. This reduction is theorized to result from resource limitations within the healthcare system, early concerns about increased bidirectional transmission in endoscopic suites, and the increased instability of patients experiencing severe respiratory failure [17,33]. However, as the literature has evolved, we have better understood that endoscopy does not increase the risk of transmission. Significant improvements in management protocols and resource allocation have also mitigated these initial concerns as substantial barriers to care [2,13,23,27,28].

Given the evidence of decreased diagnostic and therapeutic endoscopic interventions throughout the pandemic, we posit that the prevalence and severity of GI hemorrhage in patients infected with SARS-CoV-2 have likely been underestimated.

A trend toward worse morbidity and mortality rates in COVID-19 patients presenting with GI hemorrhage that meets criteria for early endoscopic intervention, as indicated by the patient’s Glasgow-Blatchford Score, has also been reported [8,34]. Multiple studies have noted increased mortality rates and a higher risk of rebleeding in patients with concurrent COVID-19 GI hemorrhage [13,20,23,27]. Goyal et al. reported mortality rates of 1.6% in COVID-19 patients presenting with GI bleeding and a rebleed risk of 10% [24]. Similarly, Iqbal et al. found mortality rates of 3.5% in these patients, with a rebleed risk of 11.3% [20]. Negro et al. demonstrated a mortality rate of 13% [19]. While these findings were not statistically significant compared to non-COVID-19 patient pools, the trends were consistently higher. All authors attributed the lack of significance to the low sample sizes within their studies [19,20,24]. A significantly longer hospital stay was observed in those infected with COVID-19 than those not [20].

Ashktorab et al. analyzed 12 separate single-center studies from Western countries and demonstrated that, within their GI hemorrhage cohort, mortality was significantly higher in those who required endoscopic intervention [7]. They also reported significantly worse outcomes in patients requiring ICU-level care and those with underlying hypertension, liver disease, and malignancy [34].

Our data align with previously published studies indicating that GI hemorrhage concurrent with SARS-CoV-2 infection results in a higher mortality rate than the reported national average. We found mortality rates of 52% among ICU patients and 48% among non-ICU patients. Our ICU and non-ICU groups were well-matched regarding comorbidities and pertinent social factors. We found no significant difference in mortality between those requiring ICU-level care and those not requiring ICU care. This suggests that GI hemorrhage may be associated with increased mortality in SARS-CoV-2 patients, independent of their level of care. Conservative management may be feasible for select patients with significant respiratory compromise for whom the risks of endoscopy outweigh the benefits. However, the demonstrated increase in morbidity, mortality, and risk of hospital-acquired infections associated with prolonged hospital stays calls the use of conservative management into question. We posit that the current standard of early endoscopic intervention within 24 hours remains the appropriate management for COVID-19-infected patients who present with signs of GI hemorrhage and can safely undergo endoscopic intervention.

The major limitation of our study was the sample size, which may have restricted the statistical significance of certain findings. Additionally, these data represent results from a single center and may be influenced by the institution's specific practice patterns.

## Conclusions

GI manifestations in patients infected with SARS-CoV-2, particularly GI hemorrhage, appear to be associated with longer hospital stays, prolonged ICU admissions, and increased mortality. Further research is needed to provide insight into the role of early endoscopic intervention in mitigating the impact of GI hemorrhage on patient outcomes in COVID-19. Our single-center data demonstrated that clinically significant GI bleeding in the presence of SARS-CoV-2 infection is strongly associated with increased disease morbidity and overall mortality, regardless of the level of care.
